# Bilaterale Vaskulitis nach intravitrealer Gabe von Brolucizumab

**DOI:** 10.1007/s00347-021-01330-7

**Published:** 2021-02-17

**Authors:** L. J. Kessler, C. S. Mayer, H. S. Son, G. U. Auffarth, R. Khoramnia

**Affiliations:** Universitäts-Augenklinik Heidelberg, Im Neuenheimer Feld 400, 69120 Heidelberg, Deutschland

## Anamnese

Eine 77-jährige kaukasische Frau wurde im September 2020 mit der Verdachtsdiagnose einer intraokularen Inflammation notfallmäßig an unsere Klinik überwiesen, nachdem sie in einer Praxis am Vortag an beiden Augen die zweite intravitreale Brolucizumab-Injektion erhalten hatte. Die Patientin berichtete, dass sie bisher in der Praxis jahrelang nahezu monatlich an beiden Augen zur Behandlung der neovaskulären Makuladegeneration (nAMD) intravitreale Injektionen mit VEGF-Inhibitoren („vascular endothelial growth factor“) erhalten habe, zuletzt mit Aflibercept. Vor 4 Wochen sei das erste Mal an beiden Augen das neu zugelassene Präparat Brolucizumab (6 mg/0,05 ml) verabreicht worden. Die Patientin berichtete, dass sie zuvor auf Aflibercept gut angesprochen habe. Der behandelnde Arzt habe ihr mitgeteilt, dass mit dem neuen Präparat möglicherweise ein größerer Therapieerfolg erzielt werden könne, und ihr deswegen vorgeschlagen, auf das neue Präparat umzustellen. Die Patientin berichtete von einem initial besseren Seheindruck beidseits in den ersten Tagen nach der ersten Injektion. Zwei Wochen später habe die Patientin ein „Spinnennetz“ am linken Auge wahrgenommen. Der behandelnde Augenarzt habe ihr bei der sofort notfallmäßig erfolgten Wiedervorstellung mitgeteilt, dass dies auf eine milde Glaskörperblutung zurückzuführen sei. Zwei Tage vor dem regulären Termin für die nächste Injektion (4 Wochen nach der ersten Injektion) habe die Patientin am linken Auge eine „Gewitterwolke“ wahrgenommen. Trotz der geschilderten Symptomatik wurde extern erneut eine beidseitige Injektion von Brolucizumab verabreicht. Eine erneute Untersuchung vor der Injektion habe nicht stattgefunden. Am selben Abend habe die Patientin an beiden Augen „nichts mehr gesehen“.

## Befund

**Figure 1:**
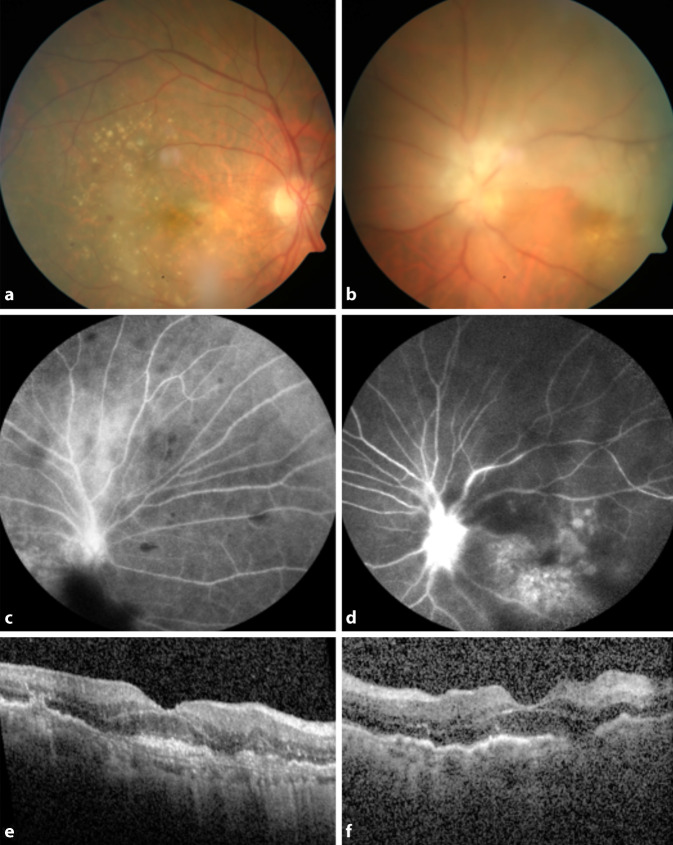

Bei der Vorstellung betrug der bestkorrigierte Visus rechts 0,1 und links 0,05. Der Visus vor der ersten Brolucizumab-Injektion ist nicht bekannt. Der Augeninnendruck betrug R/L 14 mm Hg. Bei der Untersuchung zeigten sich beidseits granulomatöse Präzipitate am Endothel, ein ausgeprägter Vorderkammerreiz sowie zahlreiche Glaskörperzellen. Trotz des verwaschenen Einblickes am linken Auge war eine Ischämie der oberen Netzhauthälfte eindeutig zu erkennen (Abb. 1a, b). Die Fluoreszenzangiographie zeigte beidseits Gefäße mit Leckage (Abb. 1c, d). In der optischen Kohärenztomografie (OCT) war ein starker Glaskörperreiz links mehr als rechts zu sehen (Abb. 1e, f).

## Diagnose

R/L intraokulare Entzündung und retinale Vaskulitis nach bilateraler Brolucizumab-Gabe, einhergehend mit einem arteriellen Gefäßverschluss (nur am linken Auge).

## Therapie und Verlauf

**Figure 2:**
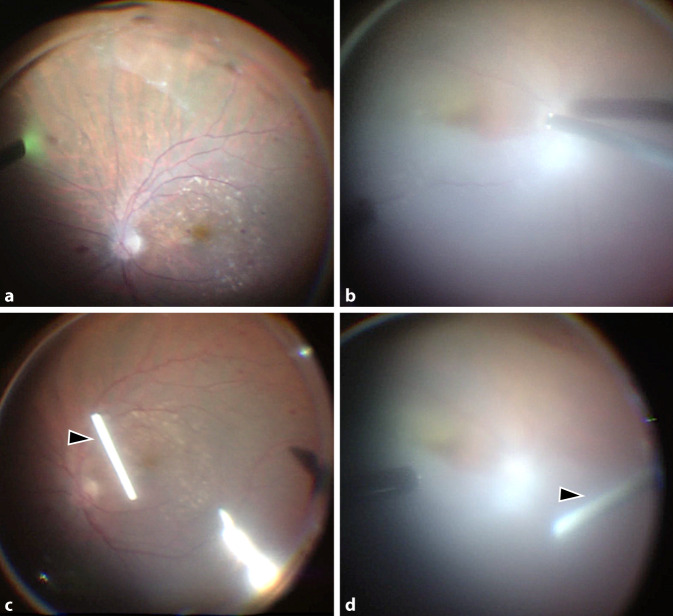

**Figure 3:**
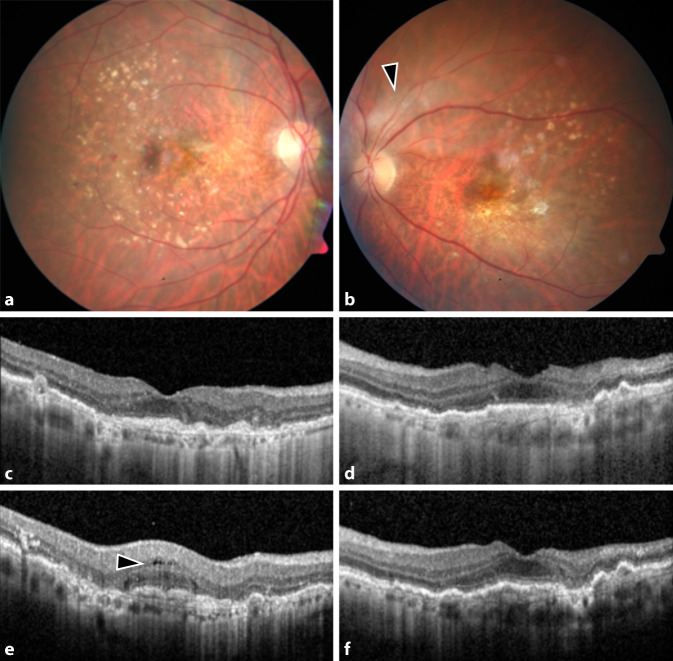

**Figure 4:**
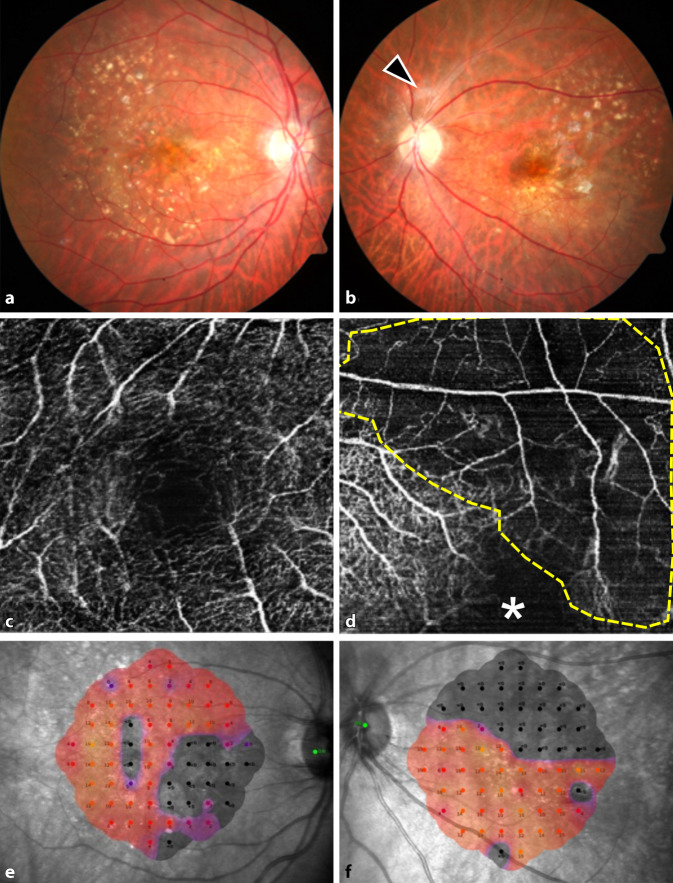

Wir erörterten mit der Patientin ausführlich die Chancen und Risiken der möglichen Therapieoptionen (intravitreale Steroidinjektion, systemische Steroidbehandlung, Pars-plana-Vitrektomie) [4, 5, 11]. Angesichts des fulminanten Verlaufes und des bereits nachgewiesenen Gefäßverschlusses am vormals besseren linken Auge entschieden wir uns auf Wunsch der Patientin für eine Maximaltherapie: Zur Entfernung des auslösenden Antigens und des inflammatorischen Materials im Glaskörperraum wurde sofort beidseits eine Vitrektomie durchgeführt. Zur Behandlung der Inflammation wurde beidseits ein Dexamethason-Implantat intravitreal implantiert (Abb. 2a–d). Die Patientin wurde außerdem zur i.v.-Therapie mit Methylprednisolon (1 g für 3 Tage, 500 mg für 2 Tage und 100 mg für 6 Tage) stationär aufgenommen. Als Lokaltherapie wurden Prednisolonacetat stündlich und Ofloxacin 4‑mal/Tag getropft. Im Glaskörperaspirat konnten keine Erreger festgestellt werden. In Zusammenschau mit der klinischen Befundbesserung unter der Kortisontherapie ist eine infektiöse Endophthalmitis differenzialdiagnostisch somit eher unwahrscheinlich. Bei Entlassung bestand beidseits ein reizloser Augenbefund mit einem bestkorrigierten Visus von 0,32 rechts und 0,5 links. Die systemische Kortisontherapie wurde bei Entlassung oralisiert und gemeinsam mit der Lokaltherapie schrittweise reduziert. Am 10. Tag nach Erstvorstellung war der intraokulare Befund beidseits reizfrei und die Makula trocken (Abb. 3a–d). Vier Wochen nach der Operation konnte am rechten Auge neue intraretinale Flüssigkeit nachgewiesen werden, sodass die Anti-VEGF-Therapie fortgesetzt wurde (Abb. 3e, f). Bei reizfreiem Augenbefund wurde Aflibercept verabreicht, da dies zuvor gut vertragen worden war. Nach der Injektion erfolgten engmaschige Kontrollen mehrmals wöchentlich. Es ergab sich kein Hinweis auf eine erneute Entzündungsreaktion. Acht Wochen nach Erstvorstellung stabilisierte sich der Visus beidseits auf 0,32. Die Patientin berichtete von einem schwarzen Balken am linken Auge, der beim Lesen störe. Funduskopisch zeigte sich links eine langstreckige Einscheidung der superioren Gefäße (Abb. 4a, b). In der OCT-Angiographie waren im superioren Makulabereich des linken Auges nur noch vereinzelte Gefäßäste zu sehen, am rechten Auge war das Kapillarnetz in der 15° × 15° OCT-Angiographie weitestgehend unauffällig (Abb. 4c, d). Die Mikroperimetrie zeigte eine Sensitivitätsminderung an beiden Augen (Abb. 4e, f). Wir planten neben der regulären Vorstellung im Rahmen der Anti-VEGF-Therapie eine Sehhilfenanpassung.

## Diskussion

Der in Deutschland seit 2020 für die Behandlung der nAMD zugelassene VEGF-Inhibitor Brolucizumab ist ein humanisiertes monoklonales Antikörperfragment mit einer Molekülmasse von 26 kDa [10]. Die Phase-3-Studien HAWK und HARRIER haben gezeigt, dass Brolucizumab in 8‑ oder 12-wöchentlichem Intervall dem festen 8‑wöchigen Injektionsschema von Aflibercept in der Behandlung der nAMD nicht unterlegen war. Ebenso waren die zentrale Netzhautdicke und die Häufigkeit von intra- oder subretinaler Flüssigkeit in der Brolucizumab-Gruppe geringer als in der Aflibercept-Gruppe [6, 10]. Bei vergleichbarem Visusverlauf und besserem anatomischem Ergebnis trotz längeren Spritzintervalls bietet Brolucizumab die Möglichkeit, die Injektionsfrequenz zu reduzieren. Dies könnte zu einer besseren Adhärenz der Patienten sowie einer Reduktion des kumulativen Infektionsrisikos beitragen.

Im Rahmen der Zulassungsstudien von Brolucizumab wurden in der Brolucizumab-Gruppe häufiger intraokulare Entzündungen beobachtet (4 % in der 6‑mg-Brolucizumab-Gruppe im Vergleich zu 1 % in der 2‑mg-Aflibercept-Gruppe). Diese wurden meistens als mild beschrieben [7, 9]. Die Anzahl der berichteten Vaskulitiden nach Brolucizumab-Injektion stieg jedoch nach der Zulassung des Präparates in den USA deutlich an. Die Inzidenz einer retinalen Vaskulitis mit und ohne Gefäßverschluss beträgt laut Post-Marketing Analysen des Herstellers (www.brolucizumab.info, Stand 23.10.20) insgesamt 14,5/10.000 Injektionen.

Es wird vermutet, dass die Brolucizumab-induzierte Vaskulopathie eine Typ-4-Hypersensitivitätsreaktion darstellt, die nicht beim erstmaligen Antigenkontakt auftritt, sondern erst verzögert im Verlauf [1, 3, 8]. Die Tatsache, dass die Patientin aber bereits nach der Erstinjektion verstärkt Floater wahrnahm, könnte darauf hindeuten, dass bei ihr möglicherweise Antikörper schon vor der Erstbehandlung vorhanden waren. Dies ist jedoch in unserem Fall nicht zu klären, da sich die Patientin nicht nach der Erstinjektion bei uns vorgestellt hatte und uns Vorbefunde leider nicht vorgelegt werden konnten. Die meisten Fallberichte schildern das Einsetzen der Symptomatik nach ca. 20 bis 30 Tagen [3].

Zum Zeitpunkt der Erstvorstellung der Patientin in unserer Klinik gab es keine Leitlinie zum Management der Vaskulitis nach einer Brolucizumab-Gabe. Baumal et al. haben aber zwischenzeitlich eine Orientierungshilfe publiziert, die auf eine individualisierte Behandlung abzielt [2]. Die Autoren fassen zusammen, dass lokale Kortikosteroide allein nur bei einer isolierten intraokularen Reizreaktion ohne Hinweis auf Vaskulitis oder Gefäßverschluss möglicherweise ausreichen können. Bei Persistenz oder Verschlechterung sowie bei Vorliegen einer Vaskulitis sollte die Kortikosteroidtherapie zügig auf die intravitreale und/oder systemische Anwendung ausgeweitet werden. In schweren Fällen mit Gefäßverschluss kann eine Vitrektomie in Erwägung gezogen werden. Bei einer okklusiven Vaskulitis wie bei unserer Patientin haben wir uns – auch auf den Wunsch der Patientin hin – für eine Maximaltherapie, bestehend aus einer Vitrektomie und Implantation eines Dexamethason-Implantats sowie einer systemischen und lokalen Kortikosteroidtherapie, entschieden. Die Kombination aus Dexamethason-Implantat und systemischer Therapie im ausschleichenden Schema erachteten wir als sinnvoll, um die Immunantwort so schnell wie möglich zu supprimieren, damit weitere Schäden vermieden werden konnten. Es gibt aber auch Fallberichte zu okklusiven Vaskulitiden, die rein konservativ behandelt wurden und ebenfalls mit einem Visusanstieg und Abklingen des Reizzustandes im Verlauf einhergingen [12]. Die klinische Erfahrung mit Brolucizumab-assoziierten intraokularen Entzündungen und/oder okklusiven Vaskulitiden ist noch sehr limitiert, sodass weitere Untersuchungen abgewartet werden müssen, bevor eine eindeutige Behandlungsempfehlung gegeben werden kann. Die rasche Einleitung der Therapie hat v. a. das Ziel weitere Schäden zu verhindern. Die Schäden, die bei einer okklusiven Vaskulitis im Bereich des Verschlussgebietes entstehen, sind leider in der Regel irreversibel.

Da die Inflammation nach intravitrealer Injektion von Brolucizumab sehr selten ist, aber in dem Fall ein schnelles Eingreifen erforderlich ist, kommt der Früherkennung eine entscheidende Bedeutung zu. Die Patienten sollten so aufgeklärt werden, dass sie relevante oder anhaltende Symptome (insbesondere [zunehmende] Mouches volantes, Schmerzen, Druckgefühl, Lichtempfindlichkeit, Visusminderung) nach intravitrealer Injektion umgehend melden, um ein rechtzeitiges Eingreifen zu erleichtern. Patienten, bei denen eine intraokulare Inflammation diagnostiziert wurde, sollten auf das Vorliegen einer begleitenden retinalen Vaskulitis und/oder eines retinalen vaskulären Verschlussereignisses untersucht werden. Die klinische Untersuchung kann durch multimodale bildgebende Verfahren ergänzt werden. Bei dem Verdacht auf eine Inflammation sollte die laufende Brolucizumab-Behandlung in jedem Falle ausgesetzt werden [2, 3]. Der Untersuchung des Fundus des Patienten in Mydriasis vor Verabreichung der Injektion kommt daher eine hohe Bedeutung zu.

Aufgrund des erhöhten Risikos einer (okklusiven) Vaskulitis bei Brolucizumab sollte das Nutzen-Risiko-Verhältnis einer zeitgleichen beidseitigen Behandlung sorgfältig abgewogen werden. Ein denkbarer Ansatz wäre, zunächst nur ein Auge mit Brolucizumab zu behandeln und das Partnerauge erst im Verlauf, wenn keine Nebenwirkungen auftreten.

## Fazit für die Praxis

- Das Auftreten von Nebenwirkungen nach Brolucizumab-Injektionen ist insgesamt sehr selten. Patienten sollten aber über eine Vaskulitis und die damit einhergehenden Symptome aufgeklärt werden.
- Vorboten einer Inflammation nach Brolucizumab-Injektionen können unspezifisch sein und sich erst Wochen nach der Injektion entwickeln. Insbesondere (zunehmende) Mouches volantes, Schmerzen, Druckgefühl, Lichtempfindlichkeit und Visusminderung sind verdächtig.
- Im Falle des Auftretens einer Inflammation besteht die Notwendigkeit einer frühzeitigen Diagnose, eines sofortigen und rechtzeitigen Eingreifens, einer intensiven Behandlung und Überwachung, um das Risiko eines Fortschreitens dieses Ereignisses zu minimieren.
- Eine Untersuchung des Fundus der Patienten in Mydriasis vor Verabreichung einer intravitrealen Injektion ist entscheidend, um eine bestehende intraokulare Inflammation auszuschließen. Unser Fall zeigt eindrücklich, dass bereits bei bestehendem Verdacht auf eine Inflammation keinesfalls eine weitere Injektion verabreicht werden sollte.
